# A Pyridazine-Based Fluorescent Probe Targeting A*β* Plaques in Alzheimer's Disease

**DOI:** 10.1155/2018/1651989

**Published:** 2018-02-27

**Authors:** Yong Dae Park, Jeum-Jong Kim, Sungbeom Lee, Chul-Hong Park, Hyoung-Woo Bai, Seung Sik Lee

**Affiliations:** ^1^Research Division for Biotechnology, Advanced Radiation Technology Institute, Korea Atomic Energy Research Institute, Jeongeup 580-185, Republic of Korea; ^2^Technology Innovation Support Team, Korea Research Institute of Chemical Technology (KRICT), Deajeon 305-600, Republic of Korea

## Abstract

Accumulation of *β*-amyloid (A*β*) plaques comprising A*β*40 and A*β*42 in the brain is the most significant factor in the pathogenesis of Alzheimer's disease (AD). Thus, the detection of A*β* plaques has increasingly attracted interest in the context of AD diagnosis. In the present study, a fluorescent pyridazine-based dye that can detect and image A*β* plaques was designed and synthesized, and its optical properties in the presence of A*β* aggregates were evaluated. An approximately 34-fold increase in emission intensity was exhibited by the fluorescent probe after binding with A*β* aggregates, for which it showed high affinity (*K*_*D*_ = 0.35 *µ*M). Moreover, the reasonable hydrophobic properties of the probe (log *P* = 2.94) allow it to penetrate the blood brain barrier (BBB). In addition, the pyridazine-based probe was used in the histological costaining of transgenic mouse (APP/PS1) brain sections to validate the selective binding of the probe to A*β* plaques. The results suggest that the pyridazine-based compound has the potential to serve as a fluorescent probe for the diagnosis of AD.

## 1. Introduction

The misfolding and aggregation of proteins cause numerous neurodegenerative diseases, such as Alzheimer's disease (AD), prion disease (PrD), and Parkinson's disease (PD) [1]. AD, one of the most common protein misfolding diseases (PMDs), is characterized by the accumulation of misfolded *β*-amyloid (A*β*) peptides and neurofibrillary tangles (NFTs) containing tau protein in the brain. A recent report revealed that the buildup of A*β* plaques in the brain plays a significant role in the pathogenesis of AD [2, 3]. Therefore, approaches to visualize A*β* deposition might prove useful for diagnosing AD and evaluating the efficacy of AD therapeutics [4–6].

Several groups have reported novel positron emission tomography (PET) imaging agents targeting A*β* plaques to diagnose AD, including BAY94-9172, FDDNP, PIB, SB-13, AV-45, and IMPY [7–13]. However, these agents are hindered by factors such as long data acquisition processes, costly equipment, exposure to radioactivity, need for proficient personnel, and comparatively poor spatial resolution [14]. Interest in monitoring the progression of AD by imaging A*β* plaques using fluorescence spectroscopy has also increased [15, 16]. Compared to nuclear imaging methods, fluorescence imaging has many advantages, including providing real-time, nonradioactive, inexpensive, and high-resolution imaging, both in vivo and ex vivo. Consequently, various fluorescent probes for imaging A*β* plaques have been developed [17–22]. An excellent fluorescent probe for A*β* plaques must meet the following requirements [18, 21, 23]: (1) selective targeting of A*β* plaques, (2) acceptable lipophilicity (log *P* value between 1 and 3), (3) high-affinity binding, (4) straightforward synthesis, and (5) a significant change in fluorescent properties upon binding to A*β* deposits.

Based on these requirements, we developed and reported fluorescent pyridazine probes targeting A*β* plaques [24]. These pyridazine probes can be used for imaging through selective binding but lack the required binding affinity for A*β* plaques. Here, we describe the optimization of pyridazine derivatives based on the conjugation of an electron acceptor with an electron donor.

**Figure 1:**
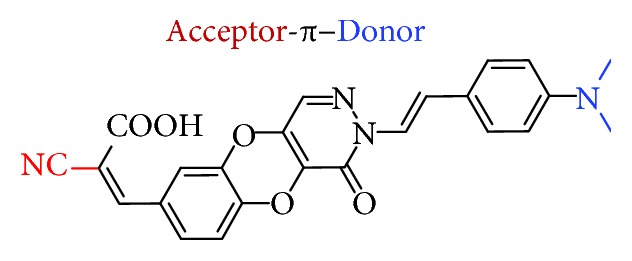
Chemical structure of fluorescent probe **3**.

To optimize these fluorescent probes, the electron-donating *p*-dimethylamino group and electron-accepting cyano group were introduced to construct a compound with a donor-*π*-acceptor structure (Figure 1). In this paper, we describe the synthesis and optical and biological properties of a cyano-based probe based on pyridazine. The ex vivo staining of A*β* plaques in APP/PS1 mice brain sections by this fluorescent probe is also presented.

## 2. Materials and Methods

### 2.1. General Experimental Methods

^1^H NMR spectra were recorded in CDCl_3_ unless otherwise noted (values in ppm) using TMS as the standard with a JNM-ECA 500 spectrometer. Low resolution mass spectra were recorded using a Varian MAT 212 mass spectrometer. IR spectra (KBr) were measured with a Bruker-Vector 22 instrument (Bruker, Bremen). Flash column chromatography was performed using silica gel (70–230 mesh). All reagent-grade chemicals were purchased from Sigma-Aldrich (St. Louis, MO, USA), and synthetic A*β*_42_ peptide was purchased from rPeptide (Bogart, GA, USA).

### 2.2. Synthesis and Characterization of Catechol Aldehyde (**2**)

A mixture of **1** (300 mg, 0.97 mmol), 3,4-dihydroxybenzaldehyde (147 mg, 1.06 mmol), and K_2_CO_3_ (293 mg, 2.12 mmol) was dissolved in DMF (20 ml) and refluxed for 24 h. After evaporating the solvent under reduced pressure, H_2_O (100 ml) and methylene chloride (50 ml) were added. The organic layer was separated and dried over MgSO_4_. The pure product (**2**) was obtained by column chromatography on silica gel using CH_2_Cl_2_ as the eluent. Yield: 89%. IR (KBr) = 3091, 2920, 2852, 1691, 1671, 1647, 1605, 1590, 1526, 1499, 1364, 1280, 1188. ^1^H NMR (CDCl_3_) = 9.83 (s, 1H), 8.01 (d, 1H, *J* = 13.75 Hz), 7.67 (s, 1H), 7.54–7.38 (m, 4H), 7.18–6.99 (m, 2H), 6.69 (d, 2H, *J* = 8.70 Hz), 2.98 (S, 6H). MS (EI) m/z 375 [M]^+^, 188, 159, 145, 117.

### 2.3. Synthesis and Characterization of Probe **3**

A mixture of **2** (100 mg, 0.27 mmol) and cyanoacetic acid (30 mg, 0.36 mmol) was vacuum dried, and CHCl_3_ (50 ml) and piperidine were added. The solution was refluxed for 15 h. Then, H_2_O (50 ml) was added. The organic layer was separated and dried over MgSO_4_. The pure product (**3**) was obtained by column chromatography on silica gel (CH_2_Cl_2_: MeOH = 6 : 1). Yield: 58%. IR (KBr) = 3398, 3091, 2922, 2853, 2211, 1651, 1632, 1603, 1524, 1503, 1363, 1335, 1277, 1187, 1163, 1125. ^1^H-NMR (CDCl_3_) = 8.04 (d, 1H, *J* = 6.86 Hz), 7.81 (d, 1H, *J* = 14.25 Hz), 7.80 (s, 1H), 7.55–7.51 (m, 2H), 7.37–7.33 (dd, 2H, *J* = 8.56, 8.52 Hz), 7.18–7.09 (m, 1H), 6.99 (d, 1H, *J* = 14.29 Hz), 6.66 (d, 2H, *J* = 7.42 Hz), 2.89 (S, 6H). MS (EI) m/z 398 [M-CO_2_]^+^, 382, 256, 145, 129, 111, 97, 83, 78, 63.

### 2.4. UV/VIS and Fluorescence Analysis

UV/VIS and fluorescence spectra were recorded and analyzed. For the UV/VIS spectra, an Infinite M200 Pro Microplate reader (Tecan, Switzerland) equipped with cells with a 1.0 cm path length was used. The scan rate was 120 nm/min. The excitation and emission *λ*_max_ values of probe **3** (10 *μ*M) were recorded with a detector (slit of 1 mm) and a data interval of 5 nm in DMF.

### 2.5. Preparation of A*β*42 Aggregates and Fluorescence Spectrum Measurement

Aggregated A*β* peptide was prepared by diluting A*β*42 to a final concentration of 100 *μ*M in PBS (pH 7.4). This solution was incubated at 200 rpm and 37°C for 3 days. The formation of A*β* fibrils was confirmed by ThT assay. The excitation and emission *λ*_max_ values of probe **3** were measured using an Infinite M200 Pro Microplate reader (Tecan, Switzerland) equipped with a detector (slit 1 mm) with a data interval of 5 nm. The scan rate was 120 nm/min. Probe **3** (10 *μ*M) was reacted with and without 20 *μ*M A*β* aggregates for 20 min in PBS at 37°C. The emission spectra and fluorescence intensity of the samples were measured. The fold increase was calculated by comparing the fluorescence intensity with and without 20 *μ*M A*β* aggregates.

### 2.6. Binding Constant (*K*_*D*_) Measurement

A 10 *μ*M solution of aggregated A*β*42 was combined with probe **3** (0.1, 0.5, 1, 2, 5, and 10 *μ*M) in PBS (pH 7.4). The solutions were incubated for 10 min at 37°C, and then their fluorescence intensity was determined at 408 nm (excitation wavelength). *K*_*D*_ was determined as described previously [25].

### 2.7. Lipophilicity (log  *P*)

Probe **3** was added to a premixed suspension containing 500 *μ*L of octanol and 500 *μ*L of PBS solution, and the resulting suspension was vortexed vigorously for 10 min and centrifuged at 3000 rpm for 5 min. Two layers separated out, and 100 *μ*L aliquots from octanol and the PBS solution layers were removed and analyzed for their fluorescence intensity. The log *P* value was calculated as the logarithm of the ratio of the fluorescence intensity in octanol versus that in PBS solution.

### 2.8. Maestro Images Analysis

An optical data study was performed using a Maestro 2.0 in vivo imaging system. The images were acquired as described previously [25]. Solutions of probe **3** (1 *μ*M) were prepared with and without 20 *μ*M A*β* aggregates in PBS. Fluorescence emission was obtained by analyzing the resulting images with commercial software (Maestro™ 2.4).

### 2.9. Histological Costaining with A*β* Antibody and Probe **3**

The brain from 12-month-old transgenic APP/PS1 mice was removed and cut into 5 *μ*m sections. The mouse brain sections were stained with probe **3** and anti-A*β* using the following method: first, the brain sections were equilibrated in PBS solution for 10 min, washed with PBS containing 0.1% Tween 20 (PBS-T) and 5% BSA for 30 min, and washed again with PBS-T supplemented with 1% BSA for 5 min 3 times. Second, the washed sections were incubated with primary antibody (rabbit anti-A*β*, 1 : 100 dilution in PBS-T supplemented with 1% BSA) overnight at 4°C, washed with PBS-T supplemented with 1% BSA 3 times, and stained with secondary antibody (Alexa 555 goat antirabbit IgG, 1 : 100 dilution in PBS-T supplemented with 1% BSA). After washing with PBS, the prestained sections were stained with 10 *µ*M probe **3** for 30 min. The stained section was washed with PBS and analyzed under an FV1000D (Olympus, Tokyo, Japan) confocal laser scanning microscope.

## 3. Results and Discussion

**Scheme 1:**
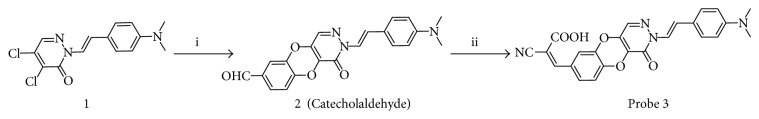
Reaction scheme for the synthesis of the pyridazine-based probe (**3**). (i) DMF, 3,4-dihydroxybenzaldehyde, K_2_CO_3_, refluxed for 24 h; (ii) CHCl_3_, cyanoacetic acid, piperidine, refluxed for 15 h.

The synthesis of probe **3** is outlined in Scheme 1. First, commercially available 3,4-dihydroxybenzaldehyde was converted to the corresponding catechol aldehyde (**2**) by reacting it with compound **1**. The Knövenagel condensation of compound **2** with cyanoacetic acid afforded the final fluorescent probe (**3**).

**Figure 2:**
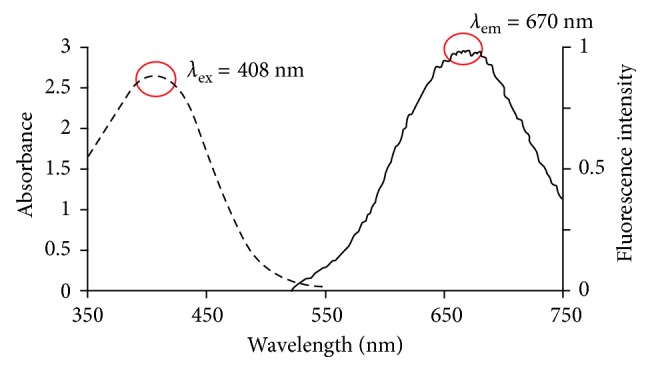
Absorbance and emission spectra of probe **3** in DMF. The maximum wavelengths in the absorbance and emission spectra are 408 nm and 670 nm, respectively.

The optical properties of the synthesized fluorescent probe (**3**) with aggregated A*β*42 peptides in PBS (pH 7.4) were analyzed, and the results are shown at Table 1. Probe **3** exhibited an excitation maximum at 408 nm and an emission maximum at 670 nm (Table 1 and Figure 2).

**Figure 3:**
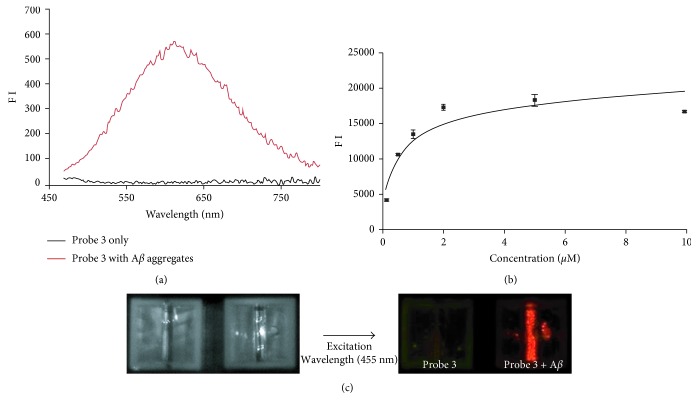
Emission spectra of probe **3** in the presence and absence of A*β* aggregates (a) and a plot of the fluorescence intensity (at *λ*_em_ = 604) as a function of the concentration of probe **3** in the presence of A*β* aggregates (10 *μ*M) in PBS (b). The apparent dissociation constant (*K*_*D*_) was 0.35 ± 0.03 *μ*M. (c) Imaging of the fluorescence intensity of probe **3** and A*β*42 aggregates using a Maestro imaging system.

To operate as a fluorescent probe targeting A*β* plaques, a compound must show a significant rise in fluorescence intensity upon binding with A*β* aggregates compared to the fluorescence intensity of free A*β* aggregates in solution [15]. Therefore, we compared the fluorescence intensity of probe **3** to the fluorescence intensity of the probe in the presence of A*β* aggregates (Figure 3(a)). As shown in Table 1, we observed a remarkable increase (35-fold) in the fluorescence intensity of probe **3** in the presence of A*β* aggregates. Additionally, the gain in fluorescence intensity was visually confirmed using a Maestro fluorescence imaging system (Figure 3(c)). This effect is due to conformational changes: When the probe in solution with A*β* aggregates is in the unbound state, free rotation through a single bond is permitted, whereas upon binding to A*β* aggregates, the probe exhibits a significant increase in fluorescence intensity due to restricted movement [26]. The binding of probe **3** to A*β* aggregates was also accompanied by a blueshift in the emission spectrum [15]. The emission wavelength of probe **3** exhibited significant blueshifts (66 nm, Table 1), indicating that probe **3** likely intercalated into the hydrophobic pocket of the A*β* aggregates. This result suggested that probe **3** could be “turned on” via an increase in fluorescence intensity and a blueshift in its emission wavelength upon interacting with A*β* aggregates.

Next, we measured the apparent binding constant (*K*_*D*_) of fluorescent probe **3** to A*β* aggregates. The fluorescence intensity of solutions of probe **3** at various concentrations in the presence of A*β* aggregates was measured, revealing that the *K*_*D*_ value of probe **3** was 0.35 ± 0.03 *μ*M (Table 1 and Figure 3(b)). This binding constant was significantly higher than that of our previously reported fluorescence probe, probe **1** (1.83 ± 0.31 *μ*M) [24]. The lipophilicity (log *P*) of probe **3** was also evaluated to determine whether it could permeate through the blood brain barrier (BBB). The log *P* value of probe **3** was found to be 2.94 (Table 1), suggesting that probe **3** has desirable properties regarding BBB permeability [21].

**Figure 4:**
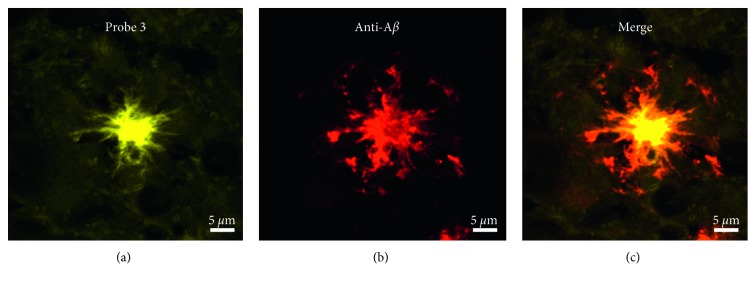
Histological double staining of 5 *µ*m double sections from the cortex of APP/PS1 mouse brains with probe **3** and anti-A*β*. All of the images were acquired at a certain excitation wavelength (anti-A*β*: 555 nm and probe **3**: 408 nm) by a confocal laser scanning microscope.

The probe developed in this paper, probe **3**, meets the requirements for a fluorescence imaging probe for AD: high fluorescence receptivity, strong binding affinity, and hydrophobicity. To assess whether fluorescent probe **3** could stain A*β* plaques in mouse brain tissue, we further evaluated the histological costaining of A*β* plaques in APP/PS1 mouse brain sections with probe **3** and anti-A*β*. A*β* plaques in the mouse brain section were identified by staining with anti-A*β* as a control. As shown in Figure 4, the brain section exposed to probe **3** exhibited significant fluorescence. Notably, the merged images showed colocalization of the areas stained with probe **3** and anti-A*β*, which demonstrates the selective targeting of A*β* plaques by probe **3**.

## 4. Conclusions

In summary, we successfully synthesized probe **3** as a novel A*β* plaque-targeting fluorescent probe by applying the concept of a donor-*π*-acceptor structure to the scaffold of a previously reported pyridazine dye, probe **1**. Probe **3** exhibited a strong fluorescence response (*F*_A*β*_/*F*_0_ > 34-fold), high affinity for A*β*42 aggregates (*K*_*D*_ = 0.35 ± 0.03 *µ*M), and sufficient hydrophobicity to penetrate the BBB (log *P* = 2.94). Furthermore, probe **3** specifically stained the A*β* plaques in APP/PS1 mouse brain sections. These results indicate probe **3** as a novel fluorescence imaging agent for the study of AD.

## Figures and Tables

**Table 1:** Fluorescence profile and *K*_*D*_ and log *P* values of probe **3** with A*β* aggregates.

Optical properties	Probe **3**
*λ*_ex_ (nm)	408
*λ*_em_ (nm)	670
*λ*_ex_/*λ*_em_ with A*β* (nm)	408/604
Fold increase with A*β*	34.92
*K*_*D*_ (mean ± SD) (*µ*M)	0.35 ± 0.03
log *P* (lipophilicity)	2.94
